# On the exoneration of Dr. William H. Stewart: debunking an urban legend

**DOI:** 10.1186/2049-9957-2-3

**Published:** 2013-02-18

**Authors:** Brad Spellberg, Bonnie Taylor-Blake

**Affiliations:** 1 Los Angeles Biomedical Research Institute at Harbor-University of California at Los Angeles (UCLA) Medical Center, Torrance, CA, USA; 2 The David Geffen School of Medicine at UCLA, Los Angeles, CA, USA; 3 University of North Carolina School of Medicine Chapel Hill, Wilmington, NC, USA

**Keywords:** William H. Stewart, Urban legend, History of antibiotics, Antibiotic development, Antibiotic crisis, Public policy

## Abstract

**Background:**

It is one of the most infamous quotes in the history of biomedicine: “It is time to close the book on infectious diseases, and declare the war against pestilence won.” Long attributed to the United States Surgeon General, Dr. William H. Stewart (1965-1969), the statement is frequently used as a foil by scientific and lay authors to underscore the ever-increasing problems of antibiotic-resistant and emerging infections. However, the primary source for the quote has never been identified.

**Methods:**

We undertook a comprehensive search of multiple databases encompassing medical literature, news articles, and congressional records to attempt to identify sources for the quote.

**Results:**

No source of the quote was identified. However, a trail of source documents was identified that clearly serves as the basis for subsequent, incorrect attribution of the quote to Dr. Stewart. In multiple source documents, Dr. Stewart made statements to the opposite effect, clearly recognizing that infectious diseases had not been conquered. The urban legend was created by a combination of lack of primary witnesses to the originating speech, misunderstanding of points made by Dr. Stewart in the speech, and increasing societal concern about emerging and re-emerging infectious diseases.

**Conclusions:**

Attribution to Dr. Stewart of a belief that it was time to close the book on infectious diseases is an urban legend; he never made any such statement. Numerous other verifiable sources, however, confirm that other people in academia adopted this belief. Dr. Stewart should no longer be cited in this regard, and should be replaced with verifiable sources.

## Multilingual abstracts

Please see Additional file 1 for translations of the abstract into the six official working languages of the United Nations.

## Background

Dr. William H. Stewart (1921-2008) was a distinguished member of the United States (US) Public Health Services Corps, having served in the first class of the Epidemic Intelligence Service officer program at the Communicable Disease Center (now the Centers for Disease Control and Prevention [CDC]), and subsequently as US Surgeon General from 1965-1969 [1]. Among his many important accomplishments were his dogged spearheading of anti-tobacco campaigns, and his aggressive attempts to end racial discrimination in health care [2].

Despite these significant achievements, history has remembered Dr. Stewart primarily for being the source of a legendary statement: “It is time to close the book on infectious diseases, and declare the war against pestilence won.” This spectacularly erroneous quote has been cited innumerable times to underscore ongoing public health problems caused by antibiotic-resistant and emerging infections. The quote was referred to in Dr. Stewart’s obituary, published in a leading medical journal in July of 2008 [3]. The persistent relevance of this quote to modern society is underscored by its citation in a 2009 Wall Street Journal article about the recent H1N1 influenza outbreak [4], and in an article regarding the deadly shiga-toxin producing *E. coli* 0104:H4 outbreak in Germany and Europe in 2011 [5].

Despite the ubiquity of its citation, there is no credible evidence that Dr. Stewart made any such statement. Before his death, the Office of the Public Health Service Historian asked Dr. Stewart if he made such a statement and his response was that he could not recall [6]. However, by following the trail of source documents back over the past 40 years, we can now reconstruct how the urban legend of Dr. Stewart’s apocryphal statement came to fruition.

## Methods

We searched electronic full-text databases for contemporaneous (late 1960s and early 1970s) and more recent attributions of these sentiments to Dr. Stewart. These databases include ProQuest Historical Newspapers (complete collection), ProQuest Direct (including ProQuest Newspapers), LexisNexis Academic, LexisNexis Congressional, JSTOR, Periodicals Archive Online (Periodicals Contents Index Full Text), Factiva, Google News, Google Books, and PubMed/Medline. Keywords for all of these search engines included various combinations of “William [H.] Stewart,” “surgeon general,” “won the war [on infectious disease],” and “close the book [on infectious disease].”

As well, Dr. Stewart’s congressional testimony, with reference to the phrases “infectious disease(s),” “close the book,” and “won the war,” was reviewed through the use of LexisNexis Congressional for the years 1966 to 1970; congressional testimony of other PHS officials were similarly scanned for such sentiments.

## Results and discussion

After many years of searching [6], only one source has been identified which contains a primary reference to Dr. Stewart’s quote [7]. The reference is to a speech given by Dr. Stewart at the 65^th^ Annual Meeting of the Association of State and Territorial Health Officers in 1967 [8]. However, a review of the actual contents of the speech revealed that it contains nothing even remotely resembling the alleged quote.To the contrary, Dr. Stewart actually said in that speech, “Warning flags are still flying in the communicable disease field…While we are engaged in taking on new duties…we cannot and must not lose sight of our traditional program responsibilities [emphasis added].” Hence, it is clear that as of 1967, Dr. Stewart was in no mood to signal the end of infectious diseases as a public health problem.

Despite an exhaustive search of multiple sources, no other primary source for Dr. Stewart’s infamous quote was identified. However, sources were identified that allow us to reconstruct the origins of the urban legend surrounding the quote.

### The seeds of an urban legend

In the aftermath of the astonishing power of antibiotics, by the 1960s the US Public Health Service was shifting its attention away from acute infections to chronic illnesses. For example, a government report published in 1968, which bears Dr. Stewart’s name on the title page, contained the following passage:

“The emphasis of epidemiologic investigation has shifted markedly in the last two decades. A decline in the interest in the infectious diseases and increase in concern with the noninfectious diseases has resulted from the change in relative importance of these categories of disease in many parts of the world, including the United States. It is also recognized that, although major tasks still remain in the improvement of control over the infectious diseases [emphasis added]…the identification of cigarette smoking as the major cause of this century’s epidemic of lung cancer…[and] chronic diseases…now constitute the predominant health problems in this country [9].”

In July of 1971, the New York Times ran an in depth commentary on a subsequent, related government report by the national Health Education Committee [10]. The reporter wrote:

American medical science has made spectacular advances in the last quarter-century, but improvements in the over-all American death rate and the infant and maternal mortality rate have leveled off…In general, Americans are almost certainly healthier and far less prey to disease than they were a few decades ago. The dread infections that used to rage through the whole communities are muted…Their retreat has been rapid since the advent of antibiotics and new vaccines after World War II…In fact, the over-all American death rate declined steadily from 1900 to about 1950. About then the improvement slowed to a virtual halt for reasons that are still obscure.

“This failure to experience a decline in mortality rates in the United States since about 1950 is little known, unexpected and extremely important,” according to Dr. William H. Stewart, former Surgeon General of the Public Health Service. He blames two factors for this new trend.

The first of these is the success of the last few decades in treating infectious disease. The degree of success was so marked and so rapid, he believes that there simply has not been room for enough further improvement to make dramatic changes in mortality.

The second factor, he says, was the emergence of chronic diseases and accidents as “the great undertone of mortality” today. These causes of death are certainly not new, but their impact was less when so many died of acute infections.”

Thus, due to: 1) the inability to further reduce mortality from infections in the aftermath of antibiotic availability; and 2) the rise of chronic illnesses as a cause of mortality, which was in large part caused by the fact that people were no longer dying at a young age of infections, Dr. Stewart and the US Public Health Service made a conscious decision to focus more on chronic illnesses and less on infectious diseases. Such a decision was not only rational, it would have been irresponsible not to pursue a change of focus in the context of the above factors. Nevertheless, the decision did not reflect a belief that infectious diseases had been “conquered” and were now irrelevant.

Within two decades, and with no published attributions in the interim, misperceptions of Dr. Stewart’s view of infections had already achieved the status of urban legend. In May of 1989, the National Institute of Allergy and Infectious Diseases (NIAID) of the National Institutes of Health (NIH) sponsored the Conference on Emerging Viruses in Washington D.C. A medical correspondent for the New York Times covered the meeting and wrote:

“The public has been generally complacent about [infectious] diseases since the development of antibiotics and certain vaccines after World War II. One example of this cited at the meeting was a 1969 speech by the Surgeon General, Dr. William H. Stewart, who assured an audience that scientists had probed most frontiers of knowledge about infectious diseases. Such attitudes, the scientists said, helped reduce the number of American researchers working on infectious diseases.Not surprisingly, the participants at the [NIAID/NIH conference] deplored this complacency” [11].

This reference in the New York Times is the first published instance we were able to identify by any of the used search engines which links Dr. Stewart to a belief that infectious diseases were no longer a compelling problem for society. No such references were identified before 1989 in any format.

A second report regarding the same NIAID conference was published in Newsday in the same month (May 1989). The article began with the following sentence. “Surgeon General William H. Stewart told Congress in 1969 that it was time to "close the book on infectious diseases," to declare the war against pestilence won and to shift national resources to such chronic disease problems as cancer and heart disease” [12]. The use of quotation marks represents the first direct attribution to Dr. Stewart of those precise words. Thus the origin of this quote can be traced directly to the 1989 conference, which Dr. Stewart did not attend. So, what was the source for the quote at the 1989 conference?

Subsequently, a text book chapter, written by one of the Conference organizers, provides the first actual citation to the alleged 1969 speech by Dr. Stewart: “The striking successes achieved with antibiotics, together with widespread application of vaccines…made many physicians and the public believe that infectious diseases were retreating and would in time be fully conquered…it had become commonplace to suggest that infectious diseases were about to become a thing of the past and that chronic, noninfectious diseases should be our major priorities” [13]. The footnote source for the statement in the text book is a personal communication to the chapter author regarding a speech given by the William Stewart as Surgeon General at Johns Hopkins University in 1969, in which he “assured his audience that infectious diseases were now of marginal interest in the United States and that we should shift our focus of attention to the chronic diseases” [14]. The originator of the personal communication was not present when Dr. Stewart gave the speech, but had heard about the contents of the speech from unspecified people who remembered the speech, which had apparently occurred during the dedication of a new building at Johns Hopkins University.

After discovering this footnoted personal communication, we requested the Johns Hopkins Medical Institutions Alan Mason Chesney Medical Archives to search for records of speeches given by Dr. Stewart at Johns Hopkins in the late 1960s. No such speech was identified in 1969. However, the Archives indeed identified a speech given by Dr. Stewart as part of the dedication of the Ernest Lyman Stebbins Building at the Johns Hopkins University School of Hygiene and Public Health on September 18, 1968 [15]. We have reviewed the content of the 1968 speech. Dr. Stewart made no statement even remotely implying the end of infections as a serious threat to public health. However, he did make statements which are clearly the seeds from which sprang the urban legend that had matured to full form by the 1989 NIAID/NIH conference:

Powerful tools have been developed to characterize these diseases in man and in society—the tools of microbiology, epidemiology, and biostatistics…means for preventing or alleviating disease in an individual or en masse have been developed and applied…and never before in man's history has a society reached such a peak of health…

This success story brings us to the here and now. And largely as a result of this success story, times have changed. The purpose of our efforts—the preservation and improvement of health—can no longer be measured on the scale of microbiology. Our exploitation of that science has just about caught up with the frontiers of public need. Clearly we cannot turn our backs on microbiology—certain notable gaps remain in our knowledge and capability, and **maintenance of a vigilant effort will always be required** [emphasis added].

But just as clearly the characterization of health in terms of microbiology and infectious disease epidemiology cannot serve as the base for our future endeavors. Rather, the moving tides of society, which we have helped to move, are compelling us to redefine our purposes in quite different terms—the terms of man's adaptation to his total environment.

Immediately we find ourselves on dark and shaky ground. This new definition of purpose rests on sciences much less comfortably exact than microbiology. We find the familiar equations of one cause—one disease disappearing into a complex of multiple causation [15].

These statements indicate that Dr. Stewart was in full grasp of the critical concepts of public health in the late 1960s. He recognized that more complex scientific tools would be necessary to reduce mortality from multi-factorial chronic illnesses than for infections. At the same time, Dr. Stewart unequivocally recognized that infections would always remain an important problem, and that “maintenance of a vigilant effort [against infections] will always be required.”

### The urban legend becomes ingrained

Two years after the 1989 NIAID conference, the origins of the quote were reinforced by a different journalist in Business Week:

“In 1969, buoyed by the defeat of smallpox, polio, and other infectious ills over the previous two decades, Surgeon General William H. Stewart declared that science had won the war on microbes. Medicine, he said, should turn to fighting chronic ailments such as cancer and heart disease. It did: From 1970 to 1975, as the budget for the National Institutes of Health more than doubled, funding for infectious disease research grew by just 20%” [16].

Again, no citation is provided for the quotation, but given the similarity to the New York Times and Newsday articles from 2 years earlier, it is highly likely that they served as the source for the Business Week article.

By the following year, the amalgamation of Dr. Stewart with the end of infections as a public health threat was completed when the following statement appeared in a book chapter written by the same journalist as the author of the 1989 Newsday article:

“In the 1960s, the world mounted a campaign against smallpox, eliminating the disease from the planet. With victory in sight, Surgeon General William H. Stewart told the U.S. Congress that it was time to "close the book on infectious diseases," declare the war against pestilence won, and shift national resources to such chronic problems as cancer and heart disease” [17].

Once again, the statement has no footnoted source in the book. An extensive review of congressional records has failed to identify any speech that Dr. Stewart gave to Congress during his stint as Surgeon General in which he claimed the end of infectious diseases had been achieved. Attribution of such a statement to him appears to reflect an erroneous conflation of concepts that Dr. Stewart appropriately discussed in his speech at Johns Hopkins University in 1968.

Subsequently, the attribution of the quotation to Dr. Stewart was burned into the consciousness of the scientific and lay communities by the same author, who wrote in a subsequent best-selling book published in 1994 that:

“By 1967 U.S. Surgeon General William H. Stewart would be so utterly convinced of imminent success that he would tell a White House gathering of state and territorial health officers that it was time to close the book on infectious diseases and shift all national attention (and dollars) to what he termed "the New Dimensions" of health: chronic diseases [9].” [7]

Note that in this follow up attribution, the same author has changed the date of the quote from 1969 to 1967, has removed the quotation marks likely reflecting an inability to identify a primary source for the statement, and has changed the venue from Congress to the meeting of the Association of State and Territorial Health Officers meeting, which is what reference #9 refers to in that book. Of course, as we have discussed above, Dr. Stewart made no such statement during that speech in 1967, the text of which is extant [8].

## Conclusion

We can therefore conclude that Dr. Stewart never promulgated the concept that infectious diseases was a dying field of research and medicine. The urban legend around his apocryphal quote resulted from initial second-hand, oral referencing of statements he made more than two decades earlier, with no successful identification of the transcript of the originating speech. Subsequent citations built upon and further inflated the initial misunderstanding of the speech. That they did so is understandable, since numerous verifiable sources confirm that the belief that infectious diseases had been successfully overcome was pervasive in biomedical circles—including among a Nobel Laureate, medical Dean, and other thought leaders—from as early as 1948, and extending all the way into the mid-1980s [18-24]. Attribution of the infamous quote reflects collateral damage to Dr. Stewart originating from conflation of the legitimate points he made, which became subsumed within the broader incorrect belief by the medical community at large that infectious diseases had been conquered. Ultimately, a string of oral communications without source documentation, followed by publication with an incorrect citation, gave rise to the full blown urban legend.

Dr. Stewart should no longer be cited as a source of the belief that infections had been conquered by modern medicine. Nor is there need to cite him in this context, because the point legitimately can continue to be made that the medical community at large believed that infectious diseases had been conquered, as evidenced by numerous other verifiable and compelling references [18-24].

## Competing interest

The authors have no conflicts of interest.

## Authors’ contributions

BS and BTB both conceived of and drafted the manuscript. BTB conducted the primary search for the source of the quote. Both authors read and approved the final manuscript.

## Supplementary Material

Additional file 1 Multilingual abstracts in the six official working languages of the United Nations. Click here for file
