# Supplementary material for: On the exoneration of Dr. William H. Stewart: debunking an urban legend
Source: Infect Dis Poverty. 2013 Feb 18;2:3. doi: 10.1186/2049-9957-2-3 (PMC3707092; doi:10.1186/2049-9957-2-3)
Supplement: Additional file 1 — Multilingual abstracts in the six official working languages of the United Nations. [file 2049-9957-2-3-S1.pdf]

Translation of the abstract into the six official working languages of the United Nations

تبرئة للدكتور ويليام أتش ستيوارت: كشف زيف أسطورة حضرية

براد سبيلبيرج و بوني تايلور بلايك

ملخص

نبذة تاريخية: حيث تعد العبارة التالية واحدة من أكثر العبارات المدانة في تاريخ الطب الحيوي : "حان وقت إغلاق سجل الأمراض المعدية و إعلان أن الحرب ضد وباء الطاعون قد تم الإنتصار فيها" وقد كانت هذه المقولة تعزي منذ زمن بعيد لوزير الصحة الأمريكي الدكتور ويليام أتش ستيوارت (1965-1969) وتستخدم هذه العبارة غالبا كغلاف بواسطة العلماء والمؤلفين من العامة لإبراز الشاكل المتزايدة لمقاومة المضادات الحيوية والإصابات الحديثة. وفي الواقع لم يعرف ابدا المصدر الرئيسي لهذه المقولة. الطرق : قمنا بإجراء بحث شامل لعدة قواعد بيانات خاصة بالأدب الطبي والمقالات الصحفية وسجلات الكونجرس محاولين تحديد مصدر هذه المقولة. النتائج: لم يتم تحديد مصدر هذه المقولة. إلا أننا قمنا بتحديد بعض مصادر المستندات والتي يمكن بوضوح أن نوصفها أنها أساس نسب هذه المقولة بطريقة خاطئة إلى الدكتور ستيوارت. وقد صرح الدكتور ستيوارت في العدسيد من المستندات بتصريحات على عكس المقولة المنسوبة إليه حيث3 أقر أن الأمراض المعدية لم يتم غزوها والإنتصار عليها. وقد نشأة هذه الاسطور الحضرية من مزيج من عدم وجود شهود أساسين لنشأة هذا الخطاب وإساءة فهم لبعض النقاط التي أوضحها دكتور ستيوارت في خطابه وزيادة الاهتمام المجتمعي بظهور وعودة ظهور الأمراض المعدية.الاستنتاجات: نسب إعتقاد أنه حان الوقت لإغلاق سجل الأمراض المعدية للدكتور ستيوارت هو أسطورة حضرية.حيث أنه لم يدلي بهذه المقولة أبدا. حيث أن العديد من المصادر التي تم التحقق من صحتها تؤكد أن هناك آخرون في المجتمع الأكاديمي آمنوا بهذا الاعتقاد. ولذا يجب عدم النقل ونسب هذه المقولة إلى الدكتور ستيوارت ويجب استبدالها بمصادر تم التحقق منها.

Translated from English version into Arabic by Mohamed Gaafar, through

TRANSLATORS WITHOUT BORDERS

Dr. William H. Stewart是无辜的：揭穿都市传说

Brad Spellberg，Bonnie Taylor-Blake

摘要

引言：在生物医学史上最臭名昭著的引言之一是，“现在是时候将传染病的书合上，并宣布已赢得抗击瘟疫的战役。”这句话长期以来曾被归诸于美国公共卫生署长（1965-1969年任职）Dr. William H. Stewart，常被作为陪饰，被科学与通俗的作者们用来强调抗生素抗性和新发感染带来的日益增多的问题。然而，该引言的出处从未得到确认。**方法**：对涵盖医学文献、新闻报道和国会记录的多个数据库进行了全面搜索，试图找出该引言的来源。**结果**：未能找到该引言源自何处。但是找到了将之归因于Dr. Stewart的、作为后来不正确引用基础的来源文件。在多个文件中，Dr. Stewart做出了与之相反的陈述，清楚地认识到传染病未被征服。这一都市传说是由缺乏主要证人的演讲、对Dr. Stewart演讲内容的误读和对于新发传染病和重现传染病日益增加的社会关注创造出来的。**结论**：Dr. Stewart从未说过现在是时候合上传染病的书了，这是一个都市传说。许多其他可核实的来源确认，学术界的其他人采用了这一观点。在这方面，Dr. Stewart不应该再被引用，而应以可证实的来源取代。

Translated from English version into Chinese by Yang Pin, through

CDC CHINESE CENTER FOR DISEASE CONTROL AND PREVENTION
NATIONAL INSTITUTE FOR PARASITIC DISEASES

La disculpation du Dr William H. Stewart : une légende urbaine démythifiée

Brad Spellberg, Bonnie Taylor-Blake

Résumé

Contexte : C'est l'une des citations les plus tristement célèbres de l'histoire de la biomédicine : « Il est temps de fermer le livre des maladies infectieuses et d'annoncer que la guerre contre la pestilence a été gagnée ». Longtemps attribuée au Dr William H. Stewart, chirurgien général américain (1965-1969), cette déclaration est fréquemment utilisée comme faire-valoir dans les publications scientifiques ou grand public visant à mettre l'accent sur les problèmes toujours croissants de l'antibiorésistance et des infections émergentes. Toutefois, la source primaire de cette citation n'a jamais été identifiée. **Méthodes :** Nous avons entrepris une recherche détaillée de bases de données multiples englobant la littérature médicale, des articles de presse et des comptes rendus de congrès pour tenter d'identifier des sources de la citation. **Résultats :** Aucune source de la citation n'a été identifiée. Toutefois, nous avons retracé une filière de documents sources qui ont manifestement servi de base à l'attribution incorrecte de la citation au Dr. Stewart. Dans de multiples documents sources, le Dr Stewart fait des déclarations du contraire, reconnaissant clairement que les maladies infectieuses n'avaient pas été éradiquées. Une combinaison de facteurs a contribué à la création de la légende urbaine : l'absence de témoins primaires du discours originel, une interprétation erronée des points avancés par le Dr Stewart dans ce discours et la montée des préoccupations sociétales sur les maladies infectieuses émergentes et ré-émergentes. **Conclusions :** Le postulat selon lequel le Dr Stewart aurait proclamé qu'il était temps de fermer le livre des maladies infectieuses relève de la légende urbaine ; il n'a jamais fait une telle déclaration. De nombreuses autres sources vérifiables confirment toutefois que cette opinion a été adoptée par d'autres dans le milieu académique. Il convient de ne plus citer le Dr Stewart à ce propos et d'utiliser à la place des sources vérifiables.

Translated from English version into French by MBernier, through

TRANSLATORS
WITHOUT BORDERS

К вопросу об оправдании доктора Уильяма Х. Стюарта: опровержение городской легенды

Брэд Спеллберг, Бонни Тейлор-Блейк

Аннотация

История: Эта цитата является одной из самых скандально известных в истории биомедицины: «Пришло время закрыть книгу инфекционных болезней. Война против эпидемий выиграна». Долгое время ее приписывали доктору Уильяму Х. Стюарту, министру здравоохранения США (1965-1969). Это высказывание часто используется как учеными, так и обывателями, чтобы на контрасте подчеркнуть важность таких проблем, как наличие устойчивых к антибиотикам инфекций и возникновение новых инфекционных болезней. Однако источник цитаты так и не был установлен.
Методы: Мы произвели тщательный поиск по многочисленным базам данных, включающих медицинскую литературу, новостные статьи и протоколы Конгресса США, с целью идентификации источника рассматриваемой цитаты.
Результаты: Источник цитаты не был обнаружен. Однако были найдены документы, которые с большой степенью вероятности послужили основой для последующего неверного приписывания данной цитаты доктору Стюарту. Согласно большому количеству оригинальных документов, доктор Стюарт делал заявления с противоположным смыслом, признавая, что борьба против инфекционных заболеваний еще не окончена. Городская легенда возникла в результате сочетания таких факторов, как отсутствие непосредственных свидетелей, непонимание заявлений доктора Стюарта во время его выступления и возрастающее беспокойство общества о вновь появляющихся и возвращающихся инфекциях.
Заключения: Приписывание доктору Стюарту мнения, что пришло время закрыть книгу инфекционных болезней, ошибочно; он никогда не делал подобных заявлений. Однако, согласно другим достоверным источникам, такого мнения придерживались некоторые другие члены академии. Таким образом, при цитировании данного заявления не следует использовать имя доктора Стюарта, а следует заменить его другими именами на основании достоверных источников.

Translated from English version into Russian by Sophia Shishatskaya, through

TRANSLATORS
WITHOUT BORDERS

La exoneración del Dr. William H. Stewart: refutando una leyenda urbana

Brad Spellberg, Bonnie Taylor-Blake

Resumen

Antecedentes: Es una de las frases más tristemente célebres de la historia de la biomedicina: "Es hora que cerremos el libro de las enfermedades infecciosas; que declaremos la victoria en la guerra contra las pestilencias". Esta declaración, por mucho tiempo atribuida al cirujano general estadounidense, Dr. William H. Stewart (1965-1969), es usada con frecuencia por los autores científicos y legos a modo de contraste, para subrayar los problemas crecientes de la resistencia a los antibióticos y las infecciones emergentes. Sin embargo, nunca se ha identificado la fuente original de esta declaración. **Métodos:** Se realizó una búsqueda exhaustiva en múltiples bases de datos, abarcando literatura médica, artículos de prensa y registros del congreso, en un intento por identificar las fuentes de la declaración. **Resultados:** No se identificó una fuente para esta declaración. Sin embargo, se identificó una huella de documentos originales que, claramente, sirven como base para la subsecuente errónea atribución de la frase al Dr. Stewart. En múltiples documentos originales, el Dr. Stewart realiza declaraciones en el sentido contrario, reconociendo, claramente, que las enfermedades infecciosas no se han conquistado. La leyenda urbana se construyó por una combinación de falta de testigos durante la declaración original, un entendimiento equivocado de los puntos realizados por el Dr. Stewart en su discurso y una creciente preocupación de la sociedad respecto a la reaparición de enfermedades infecciosas. **Conclusiones:** Atribuir al Dr. Stewart la creencia de que era tiempo de cerrar el libro de las enfermedades infecciosas, es una leyenda urbana. Él nunca hizo tal declaración. Sin embargo, numerosas fuentes verificables confirman que otras personas en la academia adoptan esta creencia. No se debería seguir citando al Dr. Stewart respecto a este asunto y se debería reemplazar por fuentes verificables.

Translated from English version into Spanish by Sjmdcl, through

TRANSLATORS WITHOUT BORDERS
